# Ivermectina subcutánea en el tratamiento de un síndrome de hiperinfección por *Strongyloides stercoralis*

**DOI:** 10.7705/biomedica.5140

**Published:** 2020-06-30

**Authors:** Diana Carolina Hennessey, Óscar Andrés Ballesteros, Diego Javier Merchán, Freddy Orlando Guevara, Diego Fernando Severiche-Bueno

**Affiliations:** 1 Departamento de Medicina Interna, Universidad El Bosque,Bogotá D.C., Colombia Universidad El Bosque Departamento de Medicina Interna Universidad El Bosque Bogotá D.C. Colombia; 2 Departamento de Medicina Crítica y Cuidado Intensivo, Universidad del Rosario, Bogotá, D.C,Colombia Departamento de Medicina Crítica y Cuidado Intensivo Universidad del Rosario BogotáD.C, Colombia; 3 Servicio de Infectología, Fundación Santafé de Bogotá,Bogotá, D.C, Colombia Fundación Santafé de Bogotá BogotáD.C Colombia; 4 Departamento de Medicina Interna,Universidad de La Sabana, Chía,Colombia Universidad de la Sabana Departamento de Medicina Interna Universidad de La Sabana ChíaColombia Colombia

**Keywords:** estrongiloidiasis/tratamiento farmacológico, ivermectina, inyecciones subcutáneas, obstrucción intestinal, inmunosupresión, Strongyloidiasis/drug therapy, ivermectin, subcutaneous injection, intestinal obstruction, immunosuppression

## Abstract

La estrongiloidiasis es una enfermedad causada por el nematodo *Strongyloides stercoralis,* endémico en las regiones rurales de los países tropicales y subtropicales. Los pacientes inmunosuprimidos tienen un mayor riesgo de infección con este parásito y pueden terminar desarrollando un síndrome de hiperinfección que conlleva un alto riesgo de muerte. En el tratamiento se utiliza la ivermectina, pero, ni en Colombia ni en el mundo, existe una presentación parenteral del medicamento para uso en humanos, lo cual es un problema en aquellos pacientes que puedan tener comprometida la absorción intestinal, como es el caso de aquellos con obstrucciones intestinales.

Se reporta el caso de un síndrome de hiperinfección por *S. stercoralis* en Colombia tratado con ivermectina subcutánea; la idea al presentarlo es incentivar los estudios de farmacocinética y farmacodinamia que analicen esta vía de administración como alternativa para el tratamiento de pacientes con riesgo de fracaso terapéutico con la vía oral.

## Abstract (en)

Strongyloidiasis is a disease caused by the nematode *Strongyloides stercoralis* that is endemic in rural regions in tropical and subtropical countries. Immunosuppressed patients have an increased risk of infection by this parasite and are at risk of developing a hyperinfection syndrome which involves a higher risk of death. The syndrome is treated with ivermectin, however, there is no parenteral presentation of this medication for human use in Colombia or the world, which is an important problem in patients who have compromised enteral absorption, for instance, those with intestinal obstructions.

We present a case of hyperinfection syndrome by *Strongyloides stercoralis* in Colombia, which was treated with subcutaneous ivermectin. Our purpose is to encourage pharmacokinetic and pharmacodynamic studies to establish this route of administration in the future as an alternative for those patients who have a high risk of therapeutic failure with the oral route.

Los pacientes inmunosuprimidos tienen un mayor riesgo de infección por gérmenes comunes y oportunistas, así como de infestaciones parasitarias. Una de ellas es la estrongiloidiasis que, en este grupo de pacientes, puede desembocar en un síndrome de hiperinfección ^1^^,^^2^.

Los pacientes que desarrollan el síndrome de hiperinfección tienen un alto riesgo de muerte y, por ello, la *Infectious Diseases Society of America* (IDSA), la *American Society of Transplantation* (AST) y los *Centers for Disease Control and Prevention* (CDC) recomiendan descartar la infestación por *Strongyloides* sp. en pacientes de áreas endémicas con síntomas gastrointestinales o eosinofilia y en lista de espera para trasplante de órgano sólido o de médula ósea, ya sea mediante la determinación de IgG por ELISA o, en caso de que la prueba no esté disponible, con un estudio parasitológico de materia fecal ^3^. Debido al curso potencialmente fatal de la enfermedad, una vez se confirma el diagnóstico, el tratamiento de primera línea se hace con ivermectina ^4^.

Sin embargo, no hay presentaciones parenterales con licencia para el uso de este medicamento en humanos, lo cual constituye un problema para aquellos que cursen con obstrucción intestinal, pues esta disminuye la absorción intestinal de la ivermectina.

A continuación, se presenta el caso de una mujer colombiana con trasplante renal que presentó un síndrome de hiperinfección y obstrucción parcial del intestino delgado. Debido al riesgo de falla terapéutica, y con su consentimiento, la paciente fue tratada con una preparación veterinaria de ivermectina, con lo cual se logró la cura de la infestación. Hasta donde se sabe por los reportes revisados, este es el primer caso documentado en Colombia y el número 24 a nivel mundial.

## Caso clínico

Se trata de una mujer de 31 años de edad con trasplante renal, bajo tratamiento con tacrolimus, prednisolona y micofenolato de mofetilo que consultó por un cuadro clínico de siete días de evolución consistente en dolor en epigastrio asociado con episodios eméticos, tos con expectoración purulenta y disnea. En el momento del ingreso, la paciente se encontraba en regular estado general con taquicardia, taquipnea y desaturación de oxígeno al medio ambiente.

**Figura 1:**
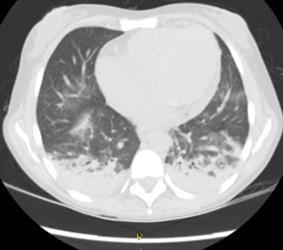
Tomografía computarizada de tórax: áreas de consolidación y atelectasias en ambos lóbulos inferiores

**Figura 2:**
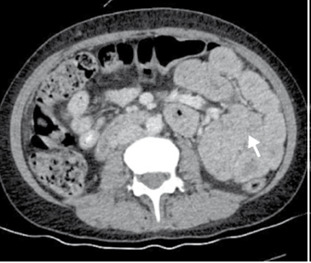
Tomografía computarizada de abdomen: dilatación del íleon distal, con engrosamiento de las paredes y signo de “fecalización” del contenido intraluminal (*small-bowel feces sign*), indicativos de posible obstrucción intestinal parcial

En los exámenes de laboratorio de ingreso, se evidenció hipoxemia moderada y, en la radiografía de tórax, opacidades en el lóbulo inferior izquierdo, por lo que se tomó una tomografía computarizada de tórax en la que se observó consolidaciones en ambos lóbulos inferiores (figura 1). Con base en estos resultados, se consideró como diagnóstico una neumonía bilateral y se inició la administración de piperacilina y tazobactam. Además, dado que persistía el dolor abdominal, se practicó una tomografía de abdomen que evidenció cambios en el íleon distal indicativos de una obstrucción intestinal parcial (figura 2).

**Figura 3:**
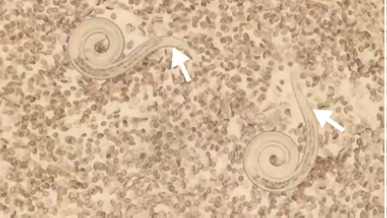
Extendido del lavado broncoalveolar: dos larvas correspondientes a *Strongyloides stercoralis*

La paciente presentó deterioro respiratorio asociado con hemoptisis, por lo que requirió intubación orotraqueal. Debido a la hemoptisis, se hizo una fibrobroncoscopia y, en el lavado broncoalveolar, se encontraron larvas de *Strongyloides* sp.; no hubo otros hallazgos (figura 3).

Dado que se trataba de una paciente inmunosuprimida, se le diagnosticó síndrome de hiperinfección por *S. stercoralis* y se inició la administración de ivermectina y albendazol por la sonda nasogástrica. Ante el riesgo de que el tratamiento fallara debido a la obstrucción intestinal, en junta médica multidisciplinaria y con el consentimiento previo de la paciente y su familia, se decidió administrarle ivermectina subcutánea en presentación para ganado vacuno, en dosis de 1,2 ml (12.000 μg o 12 mg) por vía subcutánea cada 48 horas, para un total de tres dosis. Con este tratamiento, la paciente tuvo una adecuada evolución, el síndrome de hiperinfección remitió y se le dio el egreso.

### Consideraciones éticas

Se obtuvo la aprobación y la autorización de la paciente para publicar su caso.

## Discusión

La estrongiloidiasis es una enfermedad causada por el nemátodo *S. stercoralis* que es endémico en las regiones rurales de los países tropicales y subtropicales ^5^. Se desconoce su prevalencia, pero se estima que afecta entre el 10 y el 40 % de la población mundial, llegando a alcanzar hasta el 60 % en poblaciones de bajo nivel socioeconómico, en especial, aquellas que se dedican a la agricultura ^6^. Según el estudio de Puthiyakunnon, *et al.*^7^, los países con mayor prevalencia son Namibia, Papúa Nueva Guinea, Gabón y Kenia. En cuanto a Latinoamérica, según el estudio de Buonfrate, *et al.*^8^, la prevalencia en Chile, Bolivia, Colombia y México es del 5 % y, en Argentina, Ecuador y Venezuela, del 20 %.

Entre los factores de riesgo para la hiperinfección o enfermedad diseminada, se encuentran la inmunosupresión debida a medicamentos como los corticoides, las neoplasias hematológicas y el trasplante de órgano sólido o de médula ósea ^9^. En estos pacientes, la infección puede avanzar y favorecer la migración de las larvas infectivas, alcanzando una mortalidad del 100 % ^10^.

El tratamiento de la hiperinfestación por *Strongyloides* sp. se basa en la administración de antihelmínticos, principalmente la ivermectina, sin olvidar otras opciones como el tiabendazol o el albendazol ^11^^,^^12^. En Colombia y en el mundo, sin embargo, no hay ivermectina en presentación parenteral para su uso en humanos, lo que constituye un problema en aquellos pacientes con compromiso de la absorción entérica debido, por ejemplo, a una obstrucción intestinal ^13^.

En el 2000, se publicó el primer reporte de caso a nivel mundial en el que se empleó ivermectina subcutánea en un paciente de 39 años con linfoma asociado con el HTLV-1 y con diagnóstico de hiperinfección por *Strongyloides* sp.*,* quien recibió varias dosis de 12 mg y logró la cura microbiológica ^14^.

A partir del año 2000, se han publicado, al menos, 23 casos alrededor del mundo sin que hasta la fecha haya guías de manejo o una posición clara de las sociedades científicas. Según el artículo de Barret, *et al.*^13^, las dosis diarias usadas en los reportes de casos hasta la fecha han oscilado entre 75 y 285 μg/kg (6 a 22 mg por dosis) y entre tres y 11 dosis. En 13 de los 23 casos reportados, los pacientes sobrevivieron y alcanzaron la cura microbiológica, siete alcanzaron la cura microbiológica, aunque murieron de complicaciones durante la hospitalización, y tres murieron durante el tratamiento ^13^^,^^15^.

Aunque las dosis diarias han variado, en la mayoría de los casos se usó una de 200 μg/kg, es decir, la máxima de la presentación oral ^13^^,^^15^.No obstante, no hay estudios de farmacocinética o farmacodinámica en humanos ^13^^,^^15^ que ayuden a determinar el comportamiento de la ivermectina administrada por vía subcutánea y a establecer la frecuencia de su administración, la dosis máxima tolerada y la dosis segura en humanos, entre otros datos relevantes.

## Conclusión

Este caso, el primero descrito en Colombia, debe incentivar el estudio de la farmacocinética y la farmacodinámica de la ivermectina administrada por vía subcutánea, como un primer paso para establecer esta vía de administración en el futuro y ofrecer una alternativa de tratamiento para aquellos pacientes que tienen gran riesgo de fracaso terapéutico con la vía oral.
